# Effect of PlA1/A2 glycoprotein IIIa gene polymorphism on the long-term outcome after successful coronary stenting

**DOI:** 10.1186/1477-9560-5-19

**Published:** 2007-11-16

**Authors:** Claire Le Hello, Rémy Morello, Agnès Lequerrec, Christine Duarte, John Riddell, Martial Hamon

**Affiliations:** 1 Department of Vascular Surgery, Caen Universitary Hospital, Avenue de la Côte de Nacre, 14033 Caen Cedex, France; 2 Department of Biostatistics, Caen Universitary Hospital, Avenue Georges Clémenceau, 14 000 Caen Cedex, France; 3 Department of Haemostasis, Caen Universitary Hospital, Avenue de la Côte de Nacre, 14033 Caen Cedex, France; 4 Department of Cardiology, Caen Universitary Hospital, Avenue de la Côte de Nacre, 14033 Caen Cedex, France; 5 INSERM 744, Institut Pasteur de Lille, Lille, France

## Abstract

**Aim:**

To prospectively determine the role of platelet *glycoprotein IIIa *(*GP IIIa*) gene PlA1/PlA2 polymorphism on the long-term clinical outcome in patients with coronary artery disease undergoing coronary stenting.

**Design and setting:**

Prospective observational study in the University Hospital of Caen (France).

**Patients and methods:**

1 111 symptomatic consecutive Caucasian patients treated with percutaneous coronary intervention including stent implantation underwent genotyping for GP IIIa PlA1/A2.

**Main outcome measures:**

Long-term clinical outcome in terms of the rate of major adverse cardiac events (MACE, ie death from any cause, non-fatal Q wave or non Q wave myocardial infarction, and need for coronary revascularisation) was obtained and subsequently stratified according to the GP IIIa PlA1/A2 polymorphism.

**Results:**

Three groups of patients were determined according to the GP IIIa PlA1/A2 polymorphism (71.6% had the A1/A1, 25.8% had the A1/A2 and 2.6% had the A2/A2 genotype). These three groups were comparable for all clinical characteristics including sex ratio, mean age, vascular risk factors, previous coronary events, baseline angiographic exam, indication for the percutaneous coronary intervention and drug therapy). The incidence of MACE was similar in these 3 groups of patients during a mean follow-up period of 654+/-152 days. Independent risk factors for MACE were a left ventricular ejection fraction < 40%, absence of treatment with a beta-blocker and absence of treatment with an angiotensin converting enzyme inhibitor during follow-up.

**Conclusion:**

The GP IIIa PlA1/A2 polymorphism does not influence the clinical long-term outcome in patients with symptomatic coronary disease undergoing percutaneous coronary intervention with stent implantation.

## Background

Many previous studies have shown evidence for a genetic predisposition in coronary artery disease (CAD) and some of the several tested single polymorphisms seem to be implicated [1]. The glycoprotein IIb/IIIa (GPIIb/IIIa) is a platelet membrane receptor for fibrinogen and von Willebrand factor. Glycoprotein IIIa (GPIIIa) is believed to play a central role in atherothrombosis and is a key element in platelet aggregation [2,3]. The A1/A2 GPIIIa polymorphism [4] can influence both platelet activation and aggregation [5-7] and affects post occupancy signalling by the platelet fibrinogen receptor IIb/IIIa [8]. In vitro antiaggregation by abciximab was reduced in platelets with the PlA2 polymorphism [9]. Although the Framingham Offspring Study showed heightened platelet aggregability among patients with PlA2 allele [5], the clinical impact of this polymorphism remains unclear despite many case-control studies [10-15], many observational studies [16-22] and 3 meta-analyses [23-25].

We report here our findings on the role of GP IIIa PlA1/A2 polymorphism based on long-term clinical outcome (> 18 months) of a large observational study of patients undergoing percutaneous coronary intervention (PCI) with successful coronary stenting.

## Materials and methods

### Subjects

During a period of 18 months (November 1996 to April 1998), 1 111 consecutive Caucasian patients with established CAD were prospectively enrolled in this observational study. This study complies with the declaration of Helsinki and all patients gave their written informed consent. PCI was considered to be successful if residual stenosis was < 30% by visual estimation. Diabetes was defined by either self-reporting and current use of diabetic medications or if fasting glycemia was greater than 1.26 g/l on two measurements. Dual antiplatelet therapy, using aspirin and ticlopidine, was systematically used for at least one month following coronary stenting. Criteria for non inclusion were: age under 18 years, non-Caucasian, known neoplasia, refusal to participate, inability to attend follow-up and failure to extract or amplify desoxyribonucleic acid (DNA). Among the 1111 patients, DNA could not be amplified in 66 (6.3%) patients, and 95 (9.1%) patients were lost to follow-up (for 13 of them, DNA could not be amplified). The remaining 963 patients constituted the study cohort.

### Endpoints

The primary endpoint was the composite of major adverse coronary events (MACE) including death from any cause, non-fatal Q wave or non Q wave MI, and need for coronary revascularisation by means of PCI or coronary artery bypass graft (CABG). MACE was analyzed as an aggregate endpoint and was defined before the study began. Secondary endpoints were the individual analysis of each component of the primary endpoint: total mortality, revascularization (PCI, CABG), and acute coronary syndromes.

### Genetic analysis

A blood sample was obtained at the end of the endovascular intervention after stent implantation. Genomic DNA was prepared from peripheral lymphocytes by the salt precipitation method [26]. The PlA1/A2 alleles of the *GP IIIa *gene were identified on the basis of MspI enzyme site restriction analysis after amplification of a 476 base pairs GP IIIa fragment (sens amorce 5'-ATA-AGC-TTA-GCT-ATT-GGG-AAG-TGG-TAG-GGC-CTG-3', antisens amorce 5'-CTT-CTG-ACT-CAA-GTC-CTA-ACG-3'). The *GP IIIa *gene was amplified using the polymerase chain reaction (PCR) method. Each amplification product was verified on an agarose gel. Amplification results in a 476 base pairs (bp) fragment. Digestion was obtained with Msp1 enzyme and digestion products were visualized on 4% nusieve gel: PlA1/A1 genotype results in 2 bands (279 bp and 197 bp), PlA2/A2 in 3 bands (197, 173 and 106 bp) and PlA1/A2 in 4 bands (279 bp,197 bp,173 bp and106 bp). As an additional quality-control measure, masked standards were added randomly to DNA samples received at the genotyping laboratory. All these samples were interpreted accurately.

### Follow-up

Follow-up was obtained by a questionnaire filled in during a phone call conducted by a trained physician. No scheduled time interval was specified but follow-up was at least 6 months after the successful coronary stenting procedure. Events were verified by contacting the patient's primary physician. Medical records and death certificates were also reviewed. The clinical follow-up was performed in a blind manner with respect to patient's genetic status.

### Statistical analysis

Baseline characteristics of the study population are presented as counts and percentages for categorical variables and as means for continuous variables. Differences in percentages were evaluated by the χ^2 ^test, and means by variance analysis. Validity conditions were checked for each comparison. No striking deviation from the Hardy-Weinberg equilibrium was observed in the distribution of GP IIIa PlA1/A2 polymorphism (p < 0.5). Univariate survival analysis with the Kaplan-Meier method and the log rank test were used to compare genotype groups. Significant variables (p < 0.20) in the univariate analysis were further examined in the multivariate model. Cox proportional hazards models were used in order to identify prognosis factors related to survival and PlA1/A2 GP IIIa polymorphism. The relative risks are given with 95% confidence intervals. Significance was covered by an α error of 0.05. All p values were two-tailed. All calculations were performed with SPSS Professional Statistics 13.0 (SPSS Inc, Chicago, Illinois, USA).

## Results

### Baseline characteristics of the patients

The study population (n = 963) consisted of 779 males (80.9%) and 184 females (19.1%). The mean age was 64.4 ± 11.2 years (30.8–90.9). There were no statistically significant differences in baseline clinical characteristics (vascular risk factors, medical history, indication for PCI, angiographic characteristics (left ventricular ejection fraction (LVEF) and number of diseased coronary arteries) and drug therapy at inclusion in the 3 groups of genotypes (Table 1). There were no statistically significant differences in baseline characteristics between patients with follow-up and those without follow-up. One third of patients had an angiotensin converting enzyme (ACE) inhibitor and/or a statin at inclusion. At least half of patients had a beta-blocker at inclusion. The study population was mainly treated for acute MI (28.8%) or unstable angina (37.4%).

**Table 1:** Baseline patient characteristics according to Glycoprotein IIIa (GP IIIa) polymorphism

	**GP IIIa A1/A2 genotype**			
	
	**A1/A1** **(n = 690)**	**A1/A2** **(n = 248)**	**A2/A2** **(n = 25)**	**p**
**Demographic characteristics**				
**Male sex (%)**	80.9	82.7	64	0.077
**Age (mean ± SD, years)**	64.4 ± 11.2	64.4 ± 11.4	66.2 ± 10.3	0.717
**Age ≥ 65 years (%)**	53.9	51.6	64	0.470
**Medical history**				
**Hypertension (%)**	42.0	43.1	32	0.560
**Diabetes mellitus (%)**	12.3	14.1	12	0.763
**Hypercholesterolemia (%)**	51.6	50.4	56	0.512
**Past or current smokers (%)**	50.6	53.2	40	0.417
**Body Mass Index>25 (kg/m**^2^**) (%)**	63.5	66.1	52	0.293
**Family history of CAD (%)**	27.8	29.8	24	0.755
**Prior MI (%)**	31.2	30.2	28	0.915
**Prior PCI (%)**	13.0	15.3	0	0.065
**Prior CABG (%)**	10	8.1	0	0.204
**Indication for PCI**				
**Stable angina (%)**	14.3	18.1	16	0.752
**Unstable angina (%)**	38.3	35.9	28	
**Acute MI (%)**	29.3	27.0	32	
**Post MI (%)**	11.4	12.1	16	
**Other (%)**	4.5	5.2	8	
**Angiographic characteristics**				
**LVEF < 40% (%)**	15.7	16.1	8	0.716
**Single vessel disease (%)**	45.4	42.7	32	0.576
**Two-vessel disease (%)**	32.2	35.5	36	
**Three-vessel disease (%)**	22.5	21.8	32	
**Therapy at inclusion**				
**ACE inhibitor (%)**	32.3	33.1	44	0.481
**Beta-blocker (%)**	56.1	56.0	80	0.060
**Statin (%)**	35.2	33.5	32	0.864

ACE, angiotensin converting enzyme; CABG, coronary artery bypass graft; CAD, coronary artery disease; LVEF, left ventricular ejection fraction; MI, myocardial infarction; PCI, percutaneous coronary intervention.

### Frequencies of alleles and genotypes

The frequencies of the A1 and A2 alleles were 84.5% and 15.5%, respectively. The frequencies of the A1/A1 (71.6%), A1/A2 (25.8%) and A2/A2 (2.6%) genotypes were virtually identical (Table 2) to those predicted by the Hardy-Weinberg equilibrium (Stranchan 96). There were no statistically significant differences in genotypes (p = 0.229) between patients with follow-up and those without follow-up.

**Table 2:** Observed MACE rates stratified by GP IIIa polymorphism

	**A1A1** **n = 690**	**A1A2** **n = 248**	**A2A2** **n = 25**	**p**
**MACE %**	13.6	14.1	8.0	0.730
**Death %**	4.8	8.1	0.0	0.083
**Revascularisation**				
**PCI %**	15.7	4.1	8.0	0.522
**CABG %**	2.8	3.6	4.0	0.822
**Unstable angina %**	1.7	4.0	4.2	0.095
**Acute MI %**	0.6	0.4	0.0	1.000

CABG, coronary artery bypass graft; MACE, major adverse coronary event; MI, myocardial infarction; PCI, percutaneous coronary intervention.

### Clinical follow-up of patients

**Figure 1:**
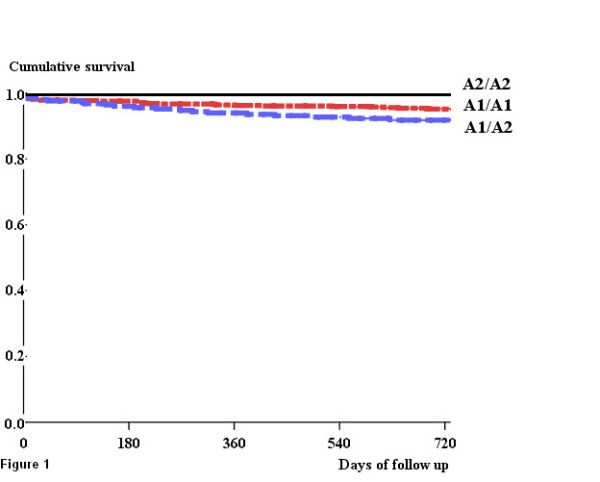
Death from any cause after revascularisation in the study population according to PlA1/A2 GP III a polymorphism (p < 0.076).

Follow-up was mainly obtained between 7 and 24 months after inclusion. Mean follow-up was 654 ± 152 days. The primary clinical end point (MACE) was reached in 13.6%, 14.1% and 8.0% of patients with the A1/A1, A1/A2 and A2/A2 genotypes respectively, with no statistically significant difference between the groups (Table 2). Table 2 details the frequencies of the components of MACE. No difference was seen in genotype distribution. Kaplan Meier analysis showed that patients of the 3 groups of genotypes had the same probability of MACE (p = 0.777), death (p = 0.076, figure 1), acute MI (p = 1.000), unstable angina (p = 0.095) and revascularisation (p = 0.522 for PCI, p = 0.822 for CABG) during the entire follow-up period. Mortality rates were 4.8%, 8.1% and 0.0% in patients with A1/A1, A1/A2 and A2/A2 genotypes respectively with no significant difference between the groups (Table 2). A1/A2 GP IIIa polymorphism was not found to be an independent predictor of MACE or death after coronary stenting. MACE and death occurred significantly more often if LVEF was < 40% (p = 0.012 and p = 0.022 respectively), if patients received no statin (p = 0.031 and p = 0.017 respectively), no ACE inhibitor (p < 0.001 for both) or no beta-blocker (p < 0.001 for both) during follow-up (Tables 3 and 4). In addition, death occurred more often in patients ≥ 65 years (p = 0.002), if patients received no beta-blocker at inclusion (p = 0.011), if no PCI was performed during follow-up (p = 0.008), and if CABG was necessary during follow-up (p = 0.047) (Table 4).

**Table 3:** Univariate analysis of MACE in the subgroups of the study population

	**MACE**		
**Subgroups**	**n**	**Survival (%)**	**p**

**Demographic characteristics**			
**Males/Females**	779/184	78.8/80.6	0.631
**Age ≥ 65/< 65 years**	516/447	79.4/78.6	0.622
**Medical history**			
**Hypertension yes/no**	405/558	77.1/80.2	0.852
**Diabetes mellitus yes/no**	123/840	73.7/79.9	0.481
**Hypercholesterolemia yes/no**	465/498	83.0/76.0	0.124
**Past or current smokers yes/no**	491/472	76.5/81.9	0.380
**BMI ≥ 25/< 25 (kg/m**^2^**)**	619/344	79.1/78.7	0.253
**Family history of MI yes/no**	272/691	78.5/79.4	0.401
**Prior MI yes/no**	297/666	82.0/77.9	0.292
**Prior PCI yes/no**	128/835	76.3/79.6	0.988
**Prior CABG yes/no**	89/874	71.3/79.9	0.564
**GP III a polymorphism**			
**A1A1/A1A2/A2A2**	690/248/25	78.3/80.6/91.7	0.777
**Coronary artery vessels diseased**			
**Single+two-/three-vessel disease**	746/217	80.2/75.3	0.109
**LVEF ≥ 40%/< 40%**	808/155	80.4/73.5	0.012
**Therapy**			
**ACE inhibitor yes/no**	316/647	69.2/81.9	0.062
**Beta-blocker yes/no**	546/417	74.9/81.3	0.262
**Statin yes/no**	334/629	76.5/79.2	0.770
**Therapy during follow-up**			
**Statin yes/no**	462/501	82.6/76.2	0.031
**ACE inhibitor yes/no**	338/625	89.3/73.3	< 0.001
**Beta-blocker yes/no**	580/383	86.5/69.3	< 0.001

ACE, angiotensin converting enzyme; BMI, body mass index; CABG, coronary artery bypass graft; LVEF, left ventricular ejection fraction; MI, myocardial infarction; PCI, percutaneous coronary intervention.

**Table 4:** Survival rates in the subgroups of the study population

	**Death**		
**Subgroups**	**n**	**Survival (%)**	**p**

**Demographic characteristics**			
**Males/Females**	779/184	94.5/93.3	0.473
**Age ≥ 65/< 65 years**	516/447	92.1/96.7	0.002
**Medical history**			
**Hypertension yes/no**	405/558	94.3/94.2	0.927
**Diabetes mellitus yes/no**	123/840	94.1/94.3	0.939
**Hypercholesterolemia yes/no**	465/498	95.8/92.8	0.069
**Past or current smoking yes/no**	491/472	95.3/93.2	0.152
**BMI ≥ 25/< 25 (kg/m**^2^**)**	619/344	95.0/93.3	0.246
**Family history of MI yes/no**	272/691	95.8/93.6	0.209
**Prior MI yes/no**	297/666	95.9/93.5	0.179
**Prior PCI yes/no**	128/835	96.0/94.0	0.396
**Prior CABG yes/no**	89/874	91.9/94.5	0.323
**GP III a polymorphism**			
**A1A1/A1A2/A2A2**	690/248/25	95.0/91.8/100.0	0.076
**PCI indication**			
**Stable angina**	152	97.3	0.572
**Unstable angina**	365	94.3	
**MI**	282	94.1	
**Post MI**	117	92.7	
**Other**	47	93.0	
**Coronary artery vessels diseased**			
**Single+two-/three-vessel disease**	746/217	94.9/92.1	0.185
**LVEF ≥ 40%/< 40%**	808/155	95.0/90.5	0.022
**Therapy**			
**ACE inhibitor yes/no**	316/647	94.4/94.1	0.886
**Beta-blocker yes/no**	546/417	96.0/92.0	0.011
**Statin yes/no**	334/629	95.9/93.3	0.114
**Revascularisation during follow-up**			
**PCI yes/no**	145/818	99.3/93.4	0.008
**CABG yes/no**	29/934	86.1/94.5	0.047
**Therapy during follow-up**			
**Statin yes/no**	462/501	96.2/92.5	0.017
**ACE inhibitor yes/no**	338/625	100.0/91.1	< 0.001
**Beta-blocker yes/no**	580/383	99.8/85.8	< 0.001

ACE, angiotensin converting enzyme; BMI, body mass index; CABG, coronary artery bypass graft; LVEF, left ventricular ejection fraction; MI, myocardial infarction; PCI, percutaneous coronary intervention.

### Multivariate analysis (Table 5)

**Table 5:** Independent risk factors for MACE and death in the study population (multivariate analysis, A1A1 (n = 690), A1A2 (n = 248), A2A2 (n = 25))

	MACE *			Death **		
	
	RR	95% CI	p	RR	95% CI	p
**LVEF < 40%**	2.07	1.37–3.13	0.001	2.81	1.53–5.16	0.001
**Follow-up**						
**Beta-blocker no/yes**	2.46	1.70–3.56	< 0.001	10.64	4.54–24.94	< 0.001
**ACE inhibitor no/yes**	2.63	1.65–4.18	< 0.001	8.91	2.74–28.98	< 0.001

* For MACE, Cox multivariable model included: hypercholesterolemia, coronary artery disease extent, LVEF, treatments during follow-up (statin and/or betablocker and/or ACE inhibitor), GP IIIa polymorphism. Other variables were not statistically significant in the univariate model.

** For death, Cox multivariable model included: age, hypercholesterolemia, past or current smoking, prior MI, family history of MI, coronary artery disease extent, LVEF, revascularisations during follow-up (PCI, CABG), treatment during follow-up (ACE inhibitor, betablocker, statin), GP IIIa polymorphism. Other variables were not statistically significant in the univariate model.

ACE, angiotensin converting enzyme; CABG, coronary artery bypass graft; CI, confidence interval; LVEF, left ventricular ejection fraction; MACE, major adverse coronary event; MI, myocardial infarction; PCI, percutaneous coronary intervention; RR, relative risk.

A multivariate analysis was performed by adjusting for several potential confounding factors. We embedded in the multivariate logistic regression model data with a p-value of less than 0.20 in the univariate analysis (clinical data, angiographic findings, genetic markers, conventional risk factors, treatments). Mortality rate was low in patients having the A2/A2 genotype. Independent risk factors for MACE after PCI were a LVEF < 40% (p = 0.001), absence of treatment with beta-blocker (p < 0.001) and absence of treatment with an ACE inhibitor (p < 0.001) during follow-up. Table 5 shows the hazard ratios and the 95% confidence intervals for these independent prognostic variables. The results were identical for death (Table 5).

## Discussion

Our prospective observational study showed that GPIIIa A1/A2 polymorphism would have no influence on long-term risk of MACE in patients having proven symptomatic CAD treated by PCI with stent implantation (bare metal stent era). This message is of clinical relevance despite recent changes in stenting procedure and adjunctive medication. Multivariate analysis showed that LVEF < 40%, and absence of treatment with beta-blocker or ACE inhibitor during follow-up were independent predictors of MACE and death in such patients. In univariate analysis, mortality was also significantly higher in patients ≥ 65 years at inclusion and those who had no revascularization during follow-up.

The role of the GP IIIa PlA1/A2 polymorphism remains controversial. It depends on the design of the studies, the characteristics of the patients included, the end-points used and the duration of follow-up. The case-control studies performed did not show any evidence of a role for the presence of the PlA2 allele in CAD, myocardial infarction (MI), premature MI or non-fatal premature MI [10-15]. Some of the observational studies suggested that PlA1/A2 polymorphism may be associated with an increased risk of CAD [16] and restenosis following coronary revascularisation [17-19]. Other observational studies suggested no association between GP IIIa polymorphism and CAD [20,21] or restenosis [22]. The first meta-analysis of relevant studies (10638 individuals) showed that the PlA2 variant of GP IIIa was not an inherited risk factor for acute coronary syndromes [23]. In the other two meta-analyses of mostly case-control studies (28503 individuals), PlA2 variant was a weak inherited risk factor for acute coronary syndromes and restenosis [24,25]. In the meta-analysis of Di Castenuovo et al., the odds ratio was higher in the 4 studies testing stent implantation (1.37, 95% CI: 1.11–1.70) than in the 2 studies testing percutaneous coronary artery dilatation (1.14, 95% CI: 0.86–1.63) [24]. This meta-analysis included case-control studies with short follow-up (≤ 6 months), and the combined clinical endpoint, MACE, was used in only one study [24,27]. Although slight, an average increase of the risk by 10% in a multifactorial disease such as CAD cannot be discounted.

The design of observational studies like ours is less controversial than that of case-control studies. In two observational studies using the clinical end point of MACE, the impact of the A2 allele was statistically significant [18,19]. The design of these 2 studies was similar to the design of our study but the follow-up was shorter (30 days and 6 months). In the first one (1 759 patients, follow-up of 30 days), A2A2 carriers experienced significantly more MACE (p = 0.06), more death or MI (p = 0.02) and more stent thrombosis (p = 0.002) [18]. In the second one (650 patients), there was no significant difference in A2 carriers for MACE after a follow-up of 6 months. Nevertheless, the number of MIs was significantly higher for A2 carriers at day 30 (p = 0.007). Restenosis rate was also higher in the A2 carriers (p = 0.06) but the difference was not significant in the multivariate analysis [19].

Our study is a prospective observational study and has the advantage of being of relatively large size (n = 963). The size of the 3 groups, the follow-up period used and the median survival time assessed at 5 years allows calculation of a power of at least 0.80 for our study to exclude an effect of the studied polymorphism. The primary endpoint used was a clinical one combining MACE (death (from any cause), non-fatal Q wave MI, proven unstable angina, and need for coronary revascularisation). The item "need for coronary revascularisation" would not have induced a survival bias because it corresponded to clinical symptomatic events (coronary angiograms were not performed systematically) and the mortality rate was similar in the different groups of patients. Furthermore the follow-up was long (mean 2.1 years) compared with previous observational studies with stent implantation [18,19]. The population in our study was a relatively high risk population: 13.6% of patients reached primary composite clinical end point and 5.5% died.

On the other hand our study has some limitations. Firstly our study was conducted in a single University Hospital characterised by regional environmental influences. The result may be different if recruitment were national. Secondly the number of A2/A2 patients was small (n = 25) explaining the inability to draw any firm conclusions for the A2/A2 genotype. It is possible that the impact of A2/A2 genotype on MACE has been underestimated. In order to confirm our result it would be necessary to enlarge this study with a bigger number of patients and particularly A2/A2 patients. Thirdly, patients were perhaps not young enough to allow us to show a statistically significant difference: mean age was 64.4 ± 11.2 years (30.8–90.9). Nevertheless the mean age was similar to that of previous studies [18,19]. Lastly, the number of DNA amplification failures and patients lost to follow-up was high. Despite the high number, this is not likely to have affected the results because clinical characteristics and genotypes (p = 0.229) of patients with follow-up and those who were lost to follow-up did not differ.

Multivariate analysis showed that LVEF < 40%, and absence of treatment with beta-blocker or ACE inhibitor during follow-up were independent predictors of MACE and death in such patients. These results are not surprising given the known beneficial effects of these drugs in CAD [28-30]. The non-prescription of these drugs may have been due to greater associated co-morbidities. In univariate analysis, mortality was significantly higher in patients ≥ 65 years at inclusion and in those who had no PCI during follow-up. The significantly higher rate of mortality for the patients who had no PCI during follow-up may be explained by silent ischemia. Patients had no systematic angiogram during follow-up and several re-stenoses may have occurred and may not have been diagnosed.

## Conclusion

Based on our results, the PlA1/A2 GP IIIa polymorphism could not be used as a risk marker of MACE in symptomatic coronary heart disease patients. Our study suggests that the A1/A2 polymorphism of GP IIIa is not a major pathophysiological factor in patients who have had coronary artery stenting.

## Authors' contributions

CLH drafted the manuscript

RM performed the statistical analysis

AL carried out the molecular genetic study

CD carried out the design of the study

JR drafted the manuscript

MH conceived of the study, participated in its design and coordination and drafted the manuscript

All authors read and approved the final manuscript

## References

[B1] Shiffman D, Rowland CM, Sninsky JJ, Devlin JJ (2006). Polymorphisms associated with coronary heart disease: better by the score. Curr Opin Mol Ther.

[B2] Nurden AT, Nurden P (1993). A review of the role of platelet membrane glycoprotein in the platelet-vessel wall interaction. Bailliere's Clin Haematol.

[B3] Lefkovits J, Plow EF, Topol EJ (1995). Platelet glycoprotein IIb/IIIa receptors in cardiovascular medicine. N Engl J Med.

[B4] Newman PJ, Derbes RS, Aster RH (1989). The human platelet alloantigens, PlA1 and PlA2 are associated with a leucine 33 to proline aminoacid polymorphism in membrane glycoprotein IIIa, and are distinguishable by DNA typing. J Clin Invest.

[B5] Feng D, Lindpaintner K, Larson MG, Rao VS, O'Donnell CJ, Lipinska I, Schmitz C, Sutherland PA, Silbershatz H, D'Agostino RB, Muller JE, Myers RH, Levy D, Tofler GH (1999). Increased platelet aggregability associated with platelet GpIIIa PlA2 polymorphism. The Framingham Offspring Study. Arterioscler Thromb Vasc Biol.

[B6] Zotz RB, Deitenbeck R, Rehfeld ISB, Maruhn-Debowski B, Guenther G, Bauer H, Winkelmann BR, Scharf RE (1997). Increased platelet sensitivity related to GPIIIa polymorphism (HPA-b/PLA2). Circulation.

[B7] Nurden AT (1995). Polymorphism of human platelet membrane glycoprotein: structure and clinical significance. Thromb Haemost.

[B8] Vijayan KV, Liu Y, Dong JF, Bray PF (2003). Enhanced activation of mitogen-activated protein kinase and myosin light chain kinase by the Pro33 polymorphism of integrin β3. Biol Chem.

[B9] Wheeler GL, Braden GA, Bray PF, Marciniak SJ, Mascelli MA, Sane DC (2002). Reduced inhibition by abciximab in platelets with the PlA2 polymorphism. Am Heart J.

[B10] Böttiger C, Kastrati A, Koch W, Mehilli J, Seidl H, Schömig K, von Beckerath N, Schömig A (2000). HPA-1 and HPA-3 polymorphisms of the platelet fibrinogen receptor and coronary artery disease and myocardial infarction. Thromb Haemost.

[B11] Bray PF, Cannon CP, Goldschmidt-Clermont P, Moyé LA, Pfeffer MA, Sacks FM, Braunwald E (2001). The platelet Pl(A2) and angiotensin-converting enzyme (ACE) D allele polymorphisms and the risk of recurrent events after acute myocardial infarction. Am J Cardiol.

[B12] Lagercrantz J, Bergman M, Lundman P, Tornvall P, Hjemdahl P, Hamsten A, Eriksson P (2003). No evidence that the PLA1/PLA2 polymorphism of the platelet glycoprotein IIIa is implicated in angiographically characterized coronary atherosclerosis and premature myocardial infarction. Blood Coagul Fibrinolysis.

[B13] Benze G, Heinrich J, Schulte H, Rust S, Nowak-Göttl U, Tataru MC, Köhler E, Assmann G, Junker R (2002). Association of the GPIa C807T and GP IIIa PlA1/A2 polymorphisms with premature myocardial infarction in men. Eur Heart J.

[B14] Mannucci M, Atherosclerosis, Thrombosis, and Vascular Biology Italian Study group (2003). No evidence of association between prothrombotic gene polymorphisms and the development of acute myocardial infarction at a young age. Circulation.

[B15] French JK, Van de Water NS, Sutton TM, Lund M, Gao W, McDowell J, Liu-Stratton Y, Pohorence J, Szymanski D, Goldschmidt-Clermont P, White HD, Browett PJ, Cooke G (2003). Potential thrombophilic mutations/polymorphisms in patients with no flow-limiting stenosis after myocardial infarction. Am Heart J.

[B16] Bojesen SE, Juul K, Schnohr P, Tybjaerg-Hansen A, Nordestgaard BG, Copenhagen City Heart Study (2003). Platelet glycoprotein IIb/IIIa PlA2/PlA2 homozygosity associated with risk of ischemic cardiovascular disease and myocardial infarction in young men. J Am Coll Cardiol.

[B17] Kastrati A, Schömig A, Seyfarth M, Koch W, Elezi S, Böttiger C, Mehilli J, Schömig K, von Beckerath N (1999). Pl^A ^polymorphism of platelet glycoprotein IIIa and risk of restenosis after coronary stent placement. Circulation.

[B18] Kastrati A, Koch W, Gawaz M, Mehilli J, Böttiger C, Schömig K, von Beckerath N, Schömig A (2000). Pl^A ^polymorphism of glycoprotein IIIa and risk of adverse events after coronary stent placement. J Am Coll Cardiol.

[B19] Walter DH, Schächinger V, Elsner M, Mach S, Dimmeler S, Auch-Schwelk W, Zeiher AM (2001). Statin therapy is associated with reduced restenosis rates after coronary stent implantation in carriers of the PlA2 allele of the glycoprotein IIIa gene. Eur Heart J.

[B20] Lopes NH, Pereira AC, Hueb W, Soares PR, Lanz JR, Gersh BJ, de Oliveira S, Cesar LA, Ramires JF, Krieger JE (2004). Effect of glycoprotein IIIa PlA2 polymorphism on outcome of patients with stable coronary artery disease and effect of smoking. Am J Cardiol.

[B21] Brscic E, Bergerone S, Gagnor A, Colajanni E, Matullo G, Scaglione L, Cassader M, Gaschino G, Di Leo M, Brusca A, Pagano GF, Piazza A, Trevi GP (2000). Acute myocardial infarction in young adults: prognostic role of the angiotensin-converting enzyme, angiotensin II type I receptor, apolipoprotein E, endothelial constitutive nitric oxide synthase, and glycoprotein IIIa genetic polymorphism at medium-term follow-up. Am Heart J.

[B22] Völzke H, Grimm R, Robinson DM, Wolff B, Schwahn C, Hertwig S, Motz W, Rettig R (2004). Candidate genetic markers and the risk of restenosis after coronary angioplasty. Clin Sci.

[B23] Zhu MM, Weedon J, Clark LT (2000). Meta-analysis of the association of platelet glycoprotein IIIa PlA1/A2 polymorphism with myocardial infarction. Am J Cardiol.

[B24] Di Castenuovo A, De Gaetano G, Donatio MB (2001). Platelet glycoprotein receptor IIIa polymorphism PlA1/A2 and coronary risk: a meta-analysis. Thromb Haemost.

[B25] Burr D, Doss H, Cooke GE, Goldschmidt-Clermont PJ (2003). A meta-analysis of studies on the association of the platelet PlA polymorphism of glycoprotein IIIa and risk of coronary heart study. Stat Med.

[B26] Miller SA, Dykes DD, Plesky HF (1988). A simple salting-out procedure for extracting DNA from human nucleated cells. Nucleic Acid Res.

[B27] Laule M, Cascorbi I, Stangl V, Bielecke C, Wernecke KD, Mrozikiewicz PM, Felix SB, Roots I, Baumann G, Stangl K (1999). A1/A2 polymorphism of glycoprotein IIIa and association with excess procedural risk for coronary catheter interventions: a case-controlled study. Lancet.

[B28] Shepherd J, Cobbe SM, Ford I, Isles CG, Lorimer AR, MacFarlane PW, McKillop JH, Packard CJ (1995). Prevention of coronary heart disease with pravastatin in men with hypercholesterolemia. West of Scotland Coronary Prevention Study Group. N Engl J Med.

[B29] Hjalmarson A, Herlitz J, Holmberg S, Rydén L, Swedberg K, Vedin A, Waagstein F, Waldenström A, Waldenström J, Wedel H, Wilhelmsen L, Wilhelmsson C (1983). The Goteborg metoprolol trial effects on mortality and morbidity in acute myocardial infarction. Circulation.

[B30] Yusuf S, Sleight P, Pogue J, Bosch J, Davies R, Dagenais G (2000). Effects of an angiotensin-converting-enzyme inhibitor, ramipril, on cardiovascular events in high-risk patients. The Heart Outcomes Prevention Evaluation Study Investigators. N Engl J Med.

